# Ogden R. Lindsley: I Followed the Idea of the Missoula Smokejumpers

**DOI:** 10.1007/s40617-024-00987-1

**Published:** 2024-08-20

**Authors:** William L. Heward, John W. Eshleman, Jonathan W. Kimball

**Affiliations:** 1 College of Education and Human Ecology, Ohio State University, Columbus, OH USA; 2 Saint Petersburg, FL USA; 3 Behavior Development Solutions, Bonita Springs, FL USA; 4 6949 Candace Place, Worthington, OH 43085 USA

**Keywords:** Accuracy addict, Errorless learning, Free operant, History of behavior analysis, Precision teaching, Rate of response, Standard Celeration Chart

## Abstract

In 1990, Ogden R. Lindsley served as guest faculty for Ohio State University’s Teleconference on applied behavior analysis. He captivated students and faculty with tales of his personal journey from experiences during World War II to studying under B. F. Skinner, and his development of precision teaching (PT) to preserve rate of response measurement in educational applications. Derived from an audio cassette recording of that seminar session, this article captures highlights of those stories and Dr. Lindsley’s discussion of topics ranging from his opinion of the open classroom movement to critiques of Sesame Street and errorless learning.

Ogden R. Lindsley devoted the greater part of his professional life to the development, exploration, and promotion of precision teaching (PT), an instructional decision-making system based on the analysis of repeated measures of learners’ rate of performance plotted on the Standard Celeration Chart. During a colorful career spanning 6 decades, Lindsley did much more than PT. He was the first to apply Skinner’s methods of free-operant conditioning with humans  (Lindsley, 1956), he coined the term *behavior therapy,* used shaping to teach a rat to press a lever twice the animal’s weight, created the dead person’s test,^1^ and played and sang country and western music.

To learn about the teaching approach Lindsley championed, see Haring et al. (2019), Kubina (2019), Pennypacker et al. (2003), Potts et al. (1993), the Special Section on Precision Teaching in *Behavior Analysis in Practice* (Bulla et al., 2021), and the Standard Celeration Society (https://www.celeration.org). To read about some of Lindsley’s other interests, accomplishments, and achievements as a pioneering scientist, see Cailkin (2023).

On November 8, 1990, Professor Lindsley served as guest faculty member for Ohio State University’s Teleconference Seminar on applied behavior analysis (Heward, 1989; Heward & Dunne, 1993). The 14 doctoral students and faculty who participated in the seminar^2^ read two articles about PT published 20 years apart in the journal *Teaching Exceptional Children* (Lindsley, 1971b, 1990a) Fig. 1.

**Fig. 1:**
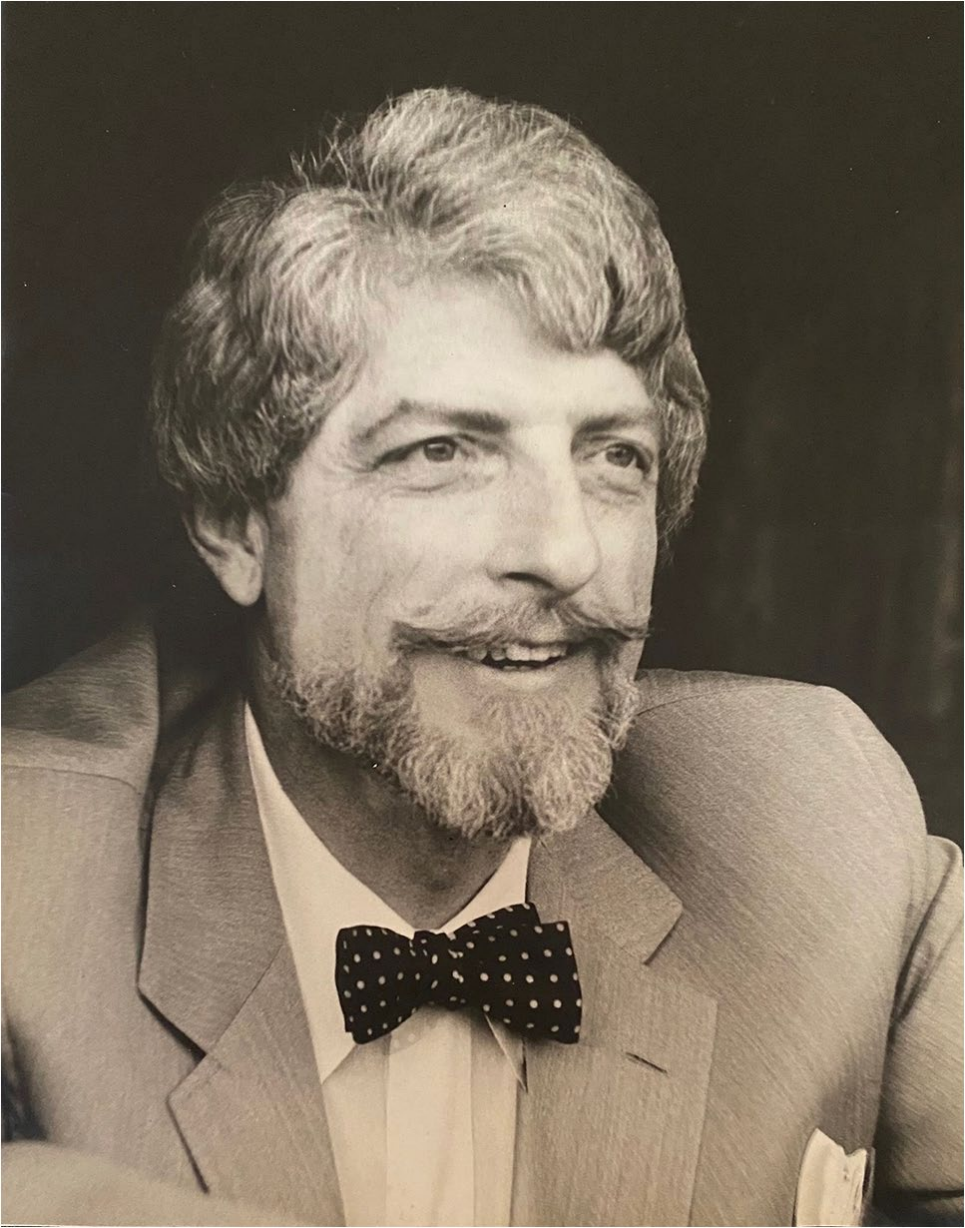
Ogden R. Lindsley (1922–2004). People often state that I developed Precision Teaching. This is incorrect. I did not develop it. It would be more accurate to say that I founded and coached it. Teachers developed it at my urging by following its founding principles. (1990, p. 10) [Photo courtesy Nancy Hughes Lindsley, photographer unknown.]

This article is derived from a transcript of an audio cassette recording of the 2-h telephone discussion with Professor Lindsley. True to his persona and preference for plain English over technical jargon and stuffy academic prose (Lindsley, 1991a), “Og” (as he preferred to be called) spoke directly and frankly, self-edited his quick-paced speech, repeated key points, and checked to make sure we were following along. He was flamboyant, entertaining, and thought provoking. He told intriguing stories, made bold predictions, and left no doubt where he stood on any topic.

## Origin Story

HEWARD: I get the privilege each week to ask our guest faculty to start things off by sharing something in the way of their personal history. What happened to Og Lindsay that led him to get so interested in behavior and to devote his life to measuring and changing it?

LINDSLEY: I was at Brown University in 1939 and 40, trying to get a Bachelor of Science in engineering. Those were troublesome times. After Pearl Harbor in 1941, the United States lowered the age requirement for aviation cadets. I’d always wanted to fly so I became an aviation cadet. I did quite well with preflight and everything but a routine flight physical detected prism divergence in my right eye. I'm a little bit cockeyed if I look up to the right above 12 diopters or something. During the flight physical a lot of the guys were erasing little red marks and stuff. The guy next in line to me said do it. I was a kid then and believed in everybody. But I thought, well, my God, be just my luck to take the nine people to their death just because I wanted my silver wings.

A few days after they washed me out as an aviation cadet, I enlisted in the Army Air Force. I ended up in the military police guarding the gate at Maxwell Field. I shot at a prisoner trying to escape. They gave me a carton of cigarettes and sent me to aviation mechanics school at Kingsley Field in Biloxi, Mississippi. I graduated top of my class in aviation mechanics and was all set to go be an engineering officer on helicopters. But because I was on detached service from the military police, I got sent back to Maxwell Field to guard the gate.

I was so angry that I went to post headquarters every day and said I was an aviation mechanic. I had an IQ of 147 and was now a corporal. I mean, what am I doing at the gate here? They finally sent me off to gunnery school and I became a gunner on a B-24 bomber. I was a top turret operator for a couple of operations. We were shot down on the way back from the Ploești oil fields [in Romania], and I became a POW in prison camp. I’m not very religious, but I kind of promised the world or the sky or something that if I got out, I'd work half the time to have fun and half the time to try to prevent this crazy thing that was going on. After the War, I went back to Brown on the GI Bill and tried to study advanced calculus. I had trouble because after 6 years in the Air Force about all I knew were four letter adjectives and the parts of a B-24 airplane and engine.

I used to study all night, trying to catch up. One morning about 4:30, I imagined the guys that got killed in the plane walking in the door and saying, “What the hell are you doing to yourself?” I would be ashamed to tell them that I was doing something I really didn't like. So, the next morning, I got out the Brown University catalog and put a checkmark next to every course I'd like to take just because of its content. If I dropped dead the day before graduation, what would I like to have in my head before I died?

I put most of the check marks next to courses in biology and psychology. I skipped an advanced calculus exam, went to the dean’s office and said I want to change to pre-med with a major in psychology. The chairman of the psych department asked if I'd ever had a course in psychology. I said, no, and he said I suggest you start with the intro course. So, I did. I got straight As and completed my bachelor’s with highest honors. I got a master's in electrophysiology. For my thesis, I used a cathode-ray oscilloscope, amplifiers, nonpolarizable microelectrodes, and all that sort of stuff to study conduction velocities in rats’ nerves.

My advisor had won notoriety with some great work recording the nerves that have to do with taste. My plan was to do the same thing with olfaction for my doctorate. I was working on my dissertation when the graduate dean dropped dead with a coronary. The acting dean was a guy with three degrees from three different universities who declared there’ll no more three degrees at Brown.

So, I went to Harvard to work with a guy who had a micro electrolysis technique that I wanted to learn. Harvard gave no credit for anything I had taken at Brown. I found myself in Psych 101 all over again and had to take maybe 120 graduate hours where usually it's like 60 or something for a doctorate. I was really angry. I got a B-minus in Boring’s exam because I missed the question, "When did psychology begin?" A B-minus was the lowest grade possible in the graduate program! Almost everybody got an A-plus-plus, an A-plus, a flat A, an A-minus or an A-minus-minus. A B-plus-plus was barely acceptable, but a B-minus could get you washed out of the program.

I was saved when Skinner asked me to assist him in the introductory course he was teaching for undergraduates. The notes for that course would become the book, *Science and Human Behavior* (Skinner, 1953/2014). Skinner asked me to train a high-jumping rat for a class demonstration. Skinner had pigeons playing ping-pong and doing different kinds of things, but he wanted some more rats to show the species generality of operant conditioning and shaping. To train the high jumping rats, I set up some ring stands and meter sticks to move the little bar up. One of the rats grabbed the end of the meter stick and started pushing it down. I thought, “Well, I'll put some weight on it with a hinge and turn him into a weightlifting rat.” In 2 weeks, Samson was lifting 250% of his own body weight.

I couldn’t believe that I had more control over a full animal in a free environment than I ever had over a nerve in a moist chamber. I would sometimes work five or six or seven Saturdays just to get one nerve firing on a cathode-ray tube. The darn things would die just before you put the electrodes on. The animal wouldn’t die, but the nerve would go dead because it had been so tortured by teasing stuff away from it and so forth.

So anyway, for a long while, I kind of compromised. I thought that I would learn the operant conditioning techniques from Skinner and then go back to electrophysiology. When you record from rat brains, you get a lot of noise from the biggest cells, which are the motor cells. And my plan was to train the rats to hold themselves still, which would clean up the brain, and then you could see the things you were studying. So, I was going to combine both techniques. I still have my electrodes, but I probably will never get around to doing that. I've been pretty much working with Skinner’s ideas ever since. That’s the story of how I got started.

HEWARD: That’s some origin story, Og! That B-minus is just still eating the hell out of you, isn't it.

LINDSLEY: No! What's eating the hell out of me is graduate students having to study equal trivia still today. I mean, there's almost no courses about the future, about where we're going, and they're going to live where we're going. What kind of sense does it make to train graduate students to be ready for 1955 or ‘65 or ‘75 or ‘85? We should be thinking about 2025.

HEWARD: At this point, let’s turn things over to the students who are excited to talk with you.

## Every Mistake I’ve Ever Made Was Because I Wasn’t Courageous Enough

OSU: Why did you name the system you developed precision teaching?

LINDSLEY: Well, I probably shouldn’t have. The reason that I went into education in 1965 was to keep rate of response alive in application. I learned, adopted, and committed to the methods of free-operant conditioning as a student of Skinner’s during the 1950s. Visitors to the laboratory I created at Harvard University to test operant conditioning with schizophrenics went back to their universities, agencies, and schools to apply our methods in their settings. But when they applied these methods to children in their classrooms, they typically neglected to use rate of response and standard recording—the hallmarks of free-operant conditioning. Instead, they adopted reward and token economy systems and recorded the percentage correct of the children's academic work, the time-honored educational measure.

In my laboratory with the schizophrenics, I had found rate of response to be any place from at least 10 and sometimes as much as 100 times more sensitive than any other index of behavior, like percent. What I mean by more sensitive is that we could pick up the effects of drugs at much lower dosages using rate of response than we could detect using percent correct as a measure of the same behavior.

The problem was that people came to my lab and said, “Jeez that’s neat, let’s do this in the classrooms." Bijou started applying behavior analysis in the classrooms at the Experimental Education Unit in Seattle. They copied my lab experiments and used cumulative recorders to record the children’s behavior in their lab studies. But in the classrooms, they used percent correct. I kept saying you’re throwing the baby out with the bath water!

Skinner said using rate of response as the basic measure of behavior and the cumulative recorder were his most important contributions.^3^ And so they took from Skinner his language and his analytical terms, even schedules of reinforcement, but they didn’t use rate to record behavior. To stop that, I followed the idea of the Missoula Montana smokejumpers. You stop a big forest fire by parachuting into the forest further than the fire has yet gone and start a little fire. Put out the side of it that is away from the real forest fire and have your little fire get going bigger and bigger and it burns out the original fire. So, I decided to parachute deep, deep into education and get teachers to have children record their own rates in the classroom. That was the plan.

In 1965, I started doing that at KU (University of Kansas) Med Center. The problem was when people started saying it was operant conditioning. And all they saw is reward. All they see when they see operant conditioning is jellybeans. They don’t see rate of response. I wanted a name that would be very different than applied behavior analysis or operant conditioning, because of the negative connotations from the folks over in the Counseling Department or the Ed Psych Department or whatever, and also from public school people and parents. They didn’t want their kids conditioned. They don’t mind them precision taught. So, I tried to pick a word that was positive and a word that suggested the detail of our recording.

And I wanted it to be an adjective, so you could have this rate recording, which we later called frequency recording, going on in counseling, going on in therapy, going on in speech path, going on in special ed, going on in social work, going on in clinical psychiatry. You could do precision psychiatry. You could do precision social work.

I realize now that I should have had more guts. Every mistake I’ve ever made was because I wasn’t courageous enough. I’ve never made mistakes being too courageous. Instead of precision teaching, I should have called it Standard Celeration Charting or something else. I should have called it what it was, rather than another thing that you have to remember and that you have to define. When you get the right name for something, you don’t need to define it. The name defines it, and precision teaching does have that weakness. But at least precision teaching includes an adjective. You can have precision learning, and you can have precision management, and precision self-management. I wanted the thing to be a noun and an adjective and a verb. So, right or wrong, that’s what I did.

I tried very hard not to be neologistic. I didn’t name the chart at all. Pennypacker wanted to write up a book, which he wrote most of, and he called it a standard *behavior* chart.^4^ Well, there are a lot of things other than behavior on that chart. It’s made so that corner to corner on all the charts is 34 degrees. And corner to corner is doubling every celeration period. That’s what’s standard about the chart. Nothing else is.

Now that’s a long, long, long answer to your question, but I think it covers the important features of it. Precision teaching is not too bad a word, but it’s not too good a word, either.^5^

## Accuracy Addiction versus Curricular Courage

*Note:* A few weeks before the teleconference session with Lindsley, several Ohio State University students attended the annual Precision Teaching conference in Boston where their appetites were whetted to learn more about the “discovered wisdom” of precision teaching. This led to several questions.

OSU: Could you explain your concept of the accuracy addict?

LINDSLEY: In the article in *Teaching Exceptional Children* (1990a), I said you have to be very careful with curricula that push accuracy—high numbers of correct responses and low errors—and that have such small steps in difficulty that children don’t make errors as they move on. The problem with that is that it doesn't produce curricular courage.

Now, if you step children up curriculum ladders with big steps, say from 100 correct per minute and 1 or 2 per minute wrong, and they jump so far up the curriculum, that corrects go down to, like 10 per minute and the errors go up to 40 or 50 errors per minute, that's what we call a crossover jaws learning picture.^6^ That gives them the courage that they can learn even when they're getting 40 wrong for every one right, or 20 wrong for everyone right. I call that *curricular courage*.

If the errors are above the corrects and learning is still going on, I call the distance between the two paths a *courage indicator*. Like if a kid's got 5 right and 80 wrong, but errors are going down and corrects are going up, then you divide 80 by 5 and you get 16. So, his *curricular courage index* is 16. That child can keep his spirits up and rapidly learn when he's wrong 16 times for every one correct.

That's what you need for creativity. That's what you need for entrepreneurship. Our public school curricula don't do that. They make kids that go into tears when their accuracy goes down to 60% or 50% correct. That's *accuracy addiction*. When you can't do something unless you get at least seven right for every three wrong. Just can't do it, it's just too hard.

Accuracy addicts are doomed to stay in the short grass. They can't be creative; they can't be entrepreneurs. The accuracy addict has to have every damned article he writes published sometime, someplace. He can't write a hundred articles and only have two published, can't have forty ideas and only three be any good.

Incidentally, the same education system doesn't insist on those accuracy requirements in the creative arts. Have you ever heard a fourth-grade music class? You're lucky to have two notes right for every fifty!

Children can handle high curricular courage. It's just the subject matter experts who can't. When you go into math, oh, boy, are you in accuracy addiction alley. There are no guesstimates. If the answer is 36 and you wrote 37, you're not even partially right. You're just plain wrong. Now more than ever we should be teaching guesstimates. We should have rapid, fluid guesstimates that you can double check with calculators and computers. We should be teaching guesstimates on exponential things. High things. What's the cube root of 4,600? You should be able to guess that to the nearest 50.

So anyway, that's accuracy addiction.

## Counter-Intuitive Discoveries

OSU: Precision teachers use a lot of different and catchy names. What does MUSIC stand for?

LINDSLEY: It’s a mnemonic for the major things that we discovered from precision teachers that are counterintuitive. They’re not facts of precision teaching, but things we discovered that shocked us, and surprised us. I used to have trouble remembering all these at workshops, so I made a memory aid for myself and started sharing it with people.^7^ MUSIC is the memory aid for the five most important counter-intuitive laws of behavior.

Whenever you look at behavior and think about it or talk about it, you’ve got to think multiply, M in place of add. The first thing we discovered was that all behavior multiplies, it doesn’t add. I think that all nature multiplies. I think the way nature multiplies is that each thing has within it growth potential. Each cow has the ability, unless she's sick or something, to make other cows. Every piece of wheat has in it little things that can make more wheat, and I think the very same thing is true of ideas and skills and all that. I think that's how our brains work, that's how our bodies work.

Why is it important? Well, if you're a teacher and you're trying to build a behavior, and you do something and you get 1 per minute…and next week you get 2 per minute. If you're thinking add and your aim is 60 per minute you'll stop what you're doing, because... it’s going to take you 58 weeks to get from 2 to 60, adding 1 a week. Now, if you think multiply, it’s 1, 2, 4, 8, 16, 32, 64, in how many weeks? Six weeks, versus 58. That's why you should think multiply, because great oaks from little acorns grow, and they grow fast.

The U in MUSIC means unique. It’s the rule rather than the exception that you need slightly different reinforcers for each person. Each person needs slightly different positions to write. But the expectation is that there is something common that will work best for everybody. The give-away is how many items do you see in the Sears-Roebuck catalog? And that’s how many items you need to reinforce buying behavior. You can’t do it otherwise. A common piece of clothing is a uniform for a prisoner. It’s aversive to have everybody wear the same clothing. But everybody has to wear the same curriculum. That’s where we are in education. We’re making prisoners out of our children by trying to use common procedures.

The S in MUSIC is specific. Behavior is very specific. If you learn to do your math standing up, you have trouble doing it sitting down. If you learn to write at a tablet on a desk you misspell at a chalkboard. You probably experienced that yourself when you started student teaching and found you couldn’t write or spell at the chalkboard. That’s because behavior is very specific. You hadn’t done chalkboard spelling yet or chalkboard writing. There is a little carry over, but it is nowhere near what people expect. They expect generalization. Now, people who have done research on generalization recently, like Don Baer at KU, say you have to teach for generalization.^8^ And the way you do it is to teach under a wide variety of conditions, which there again proves the specificity thing.

The I in MUSIC is independent. Behaviors are independent not dependent. When corrects go up, errors don’t necessarily go down. Positive feelings go up and negative feelings don’t necessarily go down. In general, when you accelerate a positive pair, like corrects and errors are a pair, and positive and negative feelings are a pair, when you accelerate the positive one—the one you want more of, the acceleration target—with about 30% of clients or students the errors will go down like you want them to. With about 30% the errors don’t change, and with about 30% the errors accelerate with corrects. It isn’t always 30%, but there's always a portion that counter celerate and a portion that co-accelerates. And we expect dependence.

The C in MUSIC is for consequence. You should think about and analyze consequence rather than cause. And educators are always looking for the causes, the antecedents. You could be in the public schools and a kid is going “whaa-whaaa-whaan whaaa whaaa, whaaa....” [Lindsley makes sound effects of a child tantrumming]. The principal comes running in and says, “What, what happened? What made him do that?” If you’re thinking cause, you say “I don’t know, I wasn’t paying attention.” If you’re thinking consequence, you say, “I don’t know. He hasn’t stopped yet.” [laughter from class].

So, that’s what MUSIC is. We have some other code words, but these are the major counter-intuitive things that we discovered using charts that we did not expect.

## Free Operant versus Controlled Operant

OSU: At the ABA [Association for Behavior Analysis] convention in May, there was a session where you, Dick Malott, and some others debated whether free operants truly exist.

LINDSLEY: I think the human being is basically a free operant creature. I think that's how we got out of the woods. There are situations where the world requires a highly controlled operant with one stimulus and you can make one and only one response. But even in many of those situations people try to make more than one response. That's why you get people saying, I love, love, love, love, LOVE YOU!! The English language only acquires one word, and they repeat that word 8 or 10 times at a high frequency to express something that the one word won't let them express. To me, that's a clear demonstration of the human free operant trying to force itself into the unnatural, controlled operant environment society has set up.

But if you were interested in the differences between free operants and controlled operants and which are best, you would set up situations in which you did both, and you record which produced more endurance, which produced greater resistance to forgetting, which produced more skill. I'm willing to bet that if a person must eventually perform in a controlled operant environment, the best training situation is free operant.^9^ These are researchable questions, not for debates among gray-headed men at ABA. These are researchable questions you kids should be working on.

When we decided to use the free operant in the classroom rather than controlled operants, we created practice sheets that had more questions or problems than any person could ever do. Those are all supported with research. When we have compared controlled operant instruction measured with percent correct with free operant instruction measured by frequency, free operant training was superior for training and practice.

## Don’t Walk to Chicago on Your Hands

OSU: The treatment of errors is an aspect of precision teaching that departs radically from current popular understanding. Following Terrace's studies published in *JEAB* [*Journal of Experimental Analysis of Behavior*] in 1963 [Terrace, 1963a, 1963b], applied researchers and practitioners have developed so-called errorless learning techniques. How do you respond to the arguments for error-free instruction?

LINDSLEY: When Terrace’s error-free discrimination papers were published, I was working with chronic schizophrenic patients. If I had to break the curriculum up into such small steps to ensure they would negotiate each and every step without error, realistically, they'd almost never get to the top of the curriculum ladder they had to climb.

I thought to myself and said to my students that Terrace provided a real neat demonstration of the power of operant conditioning. He showed operant techniques were so great that we could build behavior without any mistakes by the learner. But it's reductio ad absurdum, which is Latin for reducing something to the point of absurdity. It would be like walking to Chicago on your hands. You'll probably get on television doing it. It’s a superhuman feat and a real demonstration of your commitment to whatever purpose you were walking on your hands for. But it's an awful way to get there in a hurry.

If you're trying to get a kid to a curriculum aim in a hurry, I think insisting on a small number of errors along the way is an unnecessary impediment. Okay, that was my reaction in the early 1960s. Now, we have data that show getting from where you are to a high aim of say, 200 a minute with zero errors, is achieved more rapidly by most people starting at high errors rather than trying to get there with low errors in the beginning, much less at zero error.

Another consideration is that the world your students are going to is not a world custom tailored to be error free. The real world isn’t a highway you can walk on your hands on. In fact, if you do walk on your hands, other people will pass you. I think of an error free environment as a false environment all lined with cotton batting and temperature controlled. Nobody is ever wrong. How wonderful. But the real world isn't like that.

In fact, there are all kinds of strategies for effective use of error in the real world. One of the best strategies that I know of is to make a known error. You make an error in a certain direction and then you know how to correct for that. That's a strategy I learned when I used to mountain climb. If you camped along a river and you walk, say, straight west and now you want to get back to the camp and you got a compass, you do not go back straight east because if you make a little error and the camp isn't there, you don't know whether to go north or south to find the camp. But if you had left the camp straight west and go back east–northeast, when you come to the river, turn south, and you're sure to hit the camp.

Better to make a small known error and then correct for it. That's exactly how artillery officers in the army hit a target. They overshoot the target on purpose and then they measure the distance between where the shell fell and the target. Then they undershoot on purpose and measure that difference. It’s called zeroing in. The third shot is on target. Now imagine that with zero error. Imagine trying to teach artillery people to shoot one and only one shell and always hit the target. It's impossible. The real world isn't built that precisely. I've got a lot of other examples, but all these things convince me that it's wonderful that we demonstrated the power of operant techniques by zero error learning. But it's a very impractical thing to do if you're trying to reach an aim in a hurry. And, also, the real world these learners are going to is not programmed so carefully. They'll be in terrible trouble when they experience high errors in the real world.

## It’s Doing It, Not Watching It or Hearing It

OSU: In your opinion, what is the major problem facing public education as we move toward the twenty-first century?

LINDSLEY: The TV networks and PBS. Maybe a close second, athletics because they sap so much energy and attract all the attention.

OSU: Sesame Street is a problem?

LINDSLEY: Sesame Street is all passive, all couch potato, absolute zero performance. Mr. Rogers is a hell of a lot better. He says, go in the kitchen and get a tin can, bring it out, bang on it like I'm doing. See how I'm doing? I'm banging my can. Can you bang your can? Sesame Street doesn't even do that. Ernie and Big Bird just talk to each other.

Sesame Street has won awards for education, but it’s entertainment not education. They don't even see the difference. The evil is that some poor beginning teacher watches Sesame Street and says, "Hey, I’ll put a sock on a hand, sew on a couple of buttons, and go entertain the children." It's total waste of time. It interferes with education. It does not support it or produce it.

Let’s say Sesame Street had gone the other way and stressed the number one thing from all educators. I don't care what side the fence you are with John Dewey Maria Montessori, Fred Skinner, or Fred Keller. The number one thing is child performance is essential. It's doing it, not watching it or hearing it.

If Sesame Street decided to have the children do things, it would have been a hop, skip, and a jump before they were selling a Sesame Street School kit. To go along with Ernie and Big Bird, you had all the things in your little kit that they had up on the screen. Then it would be another skip and a jump before they sold maybe a $400 gadget with buttons that plugged into your TV. And there’d be a telephone number you could connect with and the buttons to enter responses. Now you're a hop, skip, and a jump to a real national educational network supported by public television and nonprofit foundations. Maybe I'm outspoken, but I think I'm correct.^10^

## Leaky Roofs and Satellite Dishes

OSU: There was a lot of discussion about Project Follow Through at the Boston conference. What are your opinions on why the government either suppressed or failed to provide leadership on the data from the Follow Through study [Watkins, 1988, 2017].

LINDSLEY: When I heard about Follow Through being ignored, I thought, “Oh my God. It's the largest educational experiment yet done. Thousands of kids with clearcut results, and the two most effective methods are the behavioral ones. What hope is there for us?” I said, “I'm going to talk about this scandalous situation. If we're going to ignore our research, why are we still doing it? It doesn't make any sense to have any educational research going on at all if we ignore the best.”

What happened with Project Follow Through led me to decide to not put any more energy into getting federal research and demonstration grants for precision teaching because I knew that research would be ignored too. There's no way that we could make a thing as big as Direct Instruction was in Follow Through. The Direct Instruction group touched all the right bases. They got all the grant proposals and reports in on time, they had SRA [Science Research Associates] involved in publishing the materials. And they're still ignored.

Long about then, I realized that I'd spent my life thinking that if we filled a need, that would be enough. No, you also have to fill a want. There’s a little road that runs across our ranch called Star Road. If you and I got in the pickup and drove Star Road into town, we’d notice that something like 80% of the houses have leaky roofs. And we say, let's go into the roofing business. And we do, and a couple of years go by, and we're not selling any roofs. And my partner says, "Hey, how come? What kind of mistake have we made?" We go down Star Road again and find every house with a bad roof has a satellite dish. They needed a roof, but they wanted more television. We should have showed them what they wanted, not what they needed. One reason Follow Through got ignored is because Mr. and Mrs. America don't want academic excellence, they want athletic excellence.

## Corduroy Purple Bell-bottoms

OSU: In the 1971b *Teaching Exceptional Children* article, you used the term open classroom, a popular concept at that time. It seemed that open classroom meant students were allowed to select activities and work at their own pace, lending itself to free operant kinds of behaviors. You also mentioned that in practice that most school districts interpret open classroom as simply tearing out the walls.

LINDSLEY: I was totally caught up in the open classroom idea and the '60's thing. When I conducted workshops, I wore corduroy purple bell-bottoms that hung down from my hip about four inches with a belt about three inches wide and a big peace sign for a buckle. I actually believed that the fun and the free approach would result in students learning more facts; that free people and people having fun would learn more. It didn't happen; it didn't happen. Anything learning we measured was not facilitated by the fun and the freedom. The trick is to have fun and freedom that doesn't take much time away from the actual drill and practice. I think that a classroom in the United States today has to produce all three, possibly at the expense of the facts. In other words, a teacher's got to spend some time letting the children feel that it’s their class and they're free, and they've also got to make some jokes and some fun and stuff.

HEWARD: This has been an incredible session, Og. The room is abuzz. [Students applaud.]

LINDSLEY: It was great talking to you guys. Send me a copy of the cassette tape, I’d really appreciate it.

HEWARD: Will do.

LINDSLEY: [Silence for 3 s followed by the sound of Og applauding the students.]

## Data Availability

Not applicable. No data were generated or analyzed for this article.

## Footnotes

1 In the mid-1960s, Lindsley proposed the “dead man test” to help teachers determine whether they were targeting real behaviors for measurement and change as opposed to inanimate states such as “sitting still.” According to this test, it isn’t behavior if a dead person can do it. Getting blown over by a powerful guest of wind is not behavior (a dead person would also be blown over), but lifting hands in front of one’s face, tucking and rolling, and yelling “Yikes!” while being blown over are behaviors. Critchfield (2016) provides some historical background on the dead man test and a thoughtful critique of its usefulness as a guide for measurement.

2 Students: Patricia Barbetta, Rodney Cavanaugh, Lessie Cochran, Greg Drevno, Theresa Grossi, Jeannette Lee, April D. Miller, Mary Peters, Beverly Rodenheffer, Bill Sweeney, Carolyn Talbert-Johnson. Faculty: John Cooper, John Eshleman, Bill Heward.

3 In the first chapter of Honig’s (1966) classic text, Skinner (1966) wrote a lot about his use of rate of response as the basic datum for studying behavior change. Although Skinner never says directly that rate of response was among his biggest contributions, one can intuit he considered it as such. In an interview with Skinner, Richard Evans (1968) asked: “Dr. Skinner, of all the many intriguing and provocative contributions you have made to the field of psychology... which do you feel to be most significant?” Skinner replied: “As I look back on it, it seems to me that two important things were the use of rate of responding as a basic datum and the so-called cumulative record which makes changes in rate conspicuous” (p. 103). See also, Lindsley (1991b, p. 6).

4 Pennypacker et al. (1972).

5 Throughout his career, Lindsley (1971a, 1971b, 1992) was concerned that behavior analytic terminology was confusing, A major plank of precision teaching was Lindsley’s ongoing efforts to come up with easier-to-remember, easier-to-say, less confusing terminology based on plain English. But he was not above slicing into complex terms and adopting root words as something akin to neologisms. For example, the precision teaching term *celeration* forms the root word of both *acceleration* (a word several hundred years old) and *deceleration* (coined circa 1892 by U.S. railroads to describe slowing down the movement of trains); or from adopting somewhat more complex polysyllabic words over simpler words when he felt conditions warranted, e.g., advocating the use of *frequenc*y instead of *rate* when referring to counts of events per unit of time (Lindsley, 1992).

6 Precision teachers call pairs of correct and error data paths on the SCC *learning pictures*. A common set of names distinguish learning pictures of performances that are improving (crossover, jaws, takeoff), maintaining (aim, get truckin’, rock bottom), or worsening (snowplow, landing, downhill; Lindsley, 1990a).

7 Lindsley (1977, 1990b).

8 See Baer (1981).

9 Binder (1996) discusses the use of free-operant teaching methods to build behavioral fluency.

10 For more on Sesame Street, see Lindsley (1992, pp. 24–25).
